# Multiparametric cardiac magnetic resonance imaging (CMR) for the diagnosis of Loeffler’s endocarditis: a case report

**DOI:** 10.1186/s12872-017-0492-7

**Published:** 2017-03-11

**Authors:** Mareike Gastl, Patrick Behm, Christoph Jacoby, Malte Kelm, Florian Bönner

**Affiliations:** Department of Cardiology, Pulmonology and Vascular Medicine, Heinrich-Heine University Düsseldorf, Düsseldorf, Germany

**Keywords:** CMR, Restrictive cardiomyopathy, Loeffler’s endocarditis, Heart failure, Hypereosinophilic syndrome, Fibrosis, Case report

## Abstract

**Background:**

Endocarditis parietalis fibroplastica Löfflein (EPF) is a rare form of primary restrictive cardiomyopathy with poor prognosis. It is generally caused by hypereosinophilic syndrome with eosinophilic penetration of the heart. This leads to congestive heart failure in three different stages. As a frequent manifestation of neoplastic diseases, cardiac involvement means poor prognosis.

**Case presentation:**

The present report deals with a case of EPF caused by non-specified T-cell lymphoma (T-NOS). Besides an elevated Troponin-T enzyme, the electrocardiogram and the transthoracic echocardiography did not show any characteristic results. Due to risk/benefit assessment and low thrombocyte amounts, endomyocardial biopsy and catheterization were discarded. Using cardiovascular magnetic resonance (CMR) with steady-state free precession sequences, T2-mappping, strain analysis and late gadolinium enhancement, we were able to clearly highlight cardiac involvement at different stages. These findings characterized T-NOS as a palliative situation.

**Conclusion:**

Multiparametric CMR can not only identify EPF but also characterize the patchy disease state. This provides an individual prognosis assessment. Aside from prognosis estimation, it can also be used for therapy monitoring.

## Background

Endocarditis parietalis fibroplastica (Loeffler’s disease) (EPF) is a disease with poor prognosis, first described by Loeffler in 1936. A diagnosis requires blood eosinophilia and affection of the endocardium as key steps [1]. The EPF belongs to the hypereosinophilic syndromes (HES) with rare epidemiologic data. Spry et al. showed a prevalence of 1:200000 in general population [2]. In more recent studies, the prevalence of EPF is shown from 40 to 50% among patients with HES [3]. Eosinophils must be elevated over 1.5x10^9^ eosinophils per litre for about 6 months to lead to the diagnosis of hypereosinophilia, although blood levels may lack hypereosinophilia in later stages. HES syndrome can be idiopathic or correlated to clonal interleukin 5 (IL-5) over-production of T-helper cells. Once the heart is affected, the disease passes three steps: 1. Acute necrosis followed by 2. thrombosis and 3. end stage of fibrosis [4]. The underlying pathophysiology is a degradation of cytotoxic eosinophilic proteins, which may reveal endocardial thickening, ventricular obliteration by echogenic material, suggestive of fibrosis or thrombosis, atrial dilation, restrictive pattern in echo Doppler or coronary vessel alterations [5]. Still, diagnostic process is heterogeneous and is yet to be standardized. Electrocardiogram (ECG) and transthoracic echocardiography (TTE) are unspecific and these technologies have certain restrictions (i.e. insufficient acoustic window). Endomyocardial biopsy is supposed to be the gold standard of diagnostic tools, but exerts a considerable inherent sampling error and may thus suffer from insufficient diagnostic sensitivity. Cardiovascular magnetic resonance (CMR) might be able to characterize the specific stages of the disease due to its superior native soft tissue contrast and further tissue characterisation by contrast-enhanced sequences.

## Case presentation

A 38-year-old man with a medical history of non-specified T-cell lymphoma (T-NOS) including immunosuppressive therapy regimes and a stem cell transplantation came to the emergency unit of the university hospital in Duesseldorf because of his uncontrolled nose bleeding (Table 1). Next to low thrombocyte amounts (less than 9000/μl), blood diagnostic showed an elevation of the Troponin-T enzyme up to 324 ng/l (normal cut-off at 14 ng/l). ECG revealed unspecific negative T-waves in leads II, III, aVF, V4 and V6. In TTE, only a slight hypertrophy of the interventricular septum and the left ventricular posterior wall was detected, while the global ventricular function was normal. Due to inadequate acoustic window, the quality of TTE images proved too poor for strain evaluation. The patient was transferred to the department of cardiology on suspicion of an acute myocarditis or coronary artery disease. Due to risk assessment (GRACE Score <140) and thrombocytopenia, invasive coronary angiography or endomyocardial biopsy (EMB) were initially discarded. In the meantime, a CMR examination for identification of myocardial lesions was conducted.

**Table 1:** Timeline

12/2012:	Diagnosis of non-specified T-cell lymphoma (T-NOS); Stage III B Ann Arbor
12/12 – 03/13:	CHOEP-14 protocol
03/13:	Massive progress of T-NOS (change from CHOEP to DHA, to vincristine and SDH in emergency)
04/13	Autogenic stem cell transplantation + Radiochemotheraphy (Cyclophosphamid)
06/13:	Allogenic stem cell transplantation
06/13:	Thrombosis Vena cava inferior/iliaca communis
10/13:	Remission of T-NOS
07/15:	Relapse of T-NOS
07/15 – 01/16:	IGEV-Therapy, bendamustine, DLI
01/16:	Change to everolimus
01/16:	Diagnosis of Loeffler’s endocarditis

*CHOEP-14* cyclophosphamide, doxorubicin, vincristine, etoposide and prednisone, *DHA* Docosahexaenoic acid, *SDH* Solu-Decortin, *IGEV* ifosfamide, gemcitabine, vinorelbine and prednisone, *DLI* donor lymphcyte infusion

**Fig. 1:**
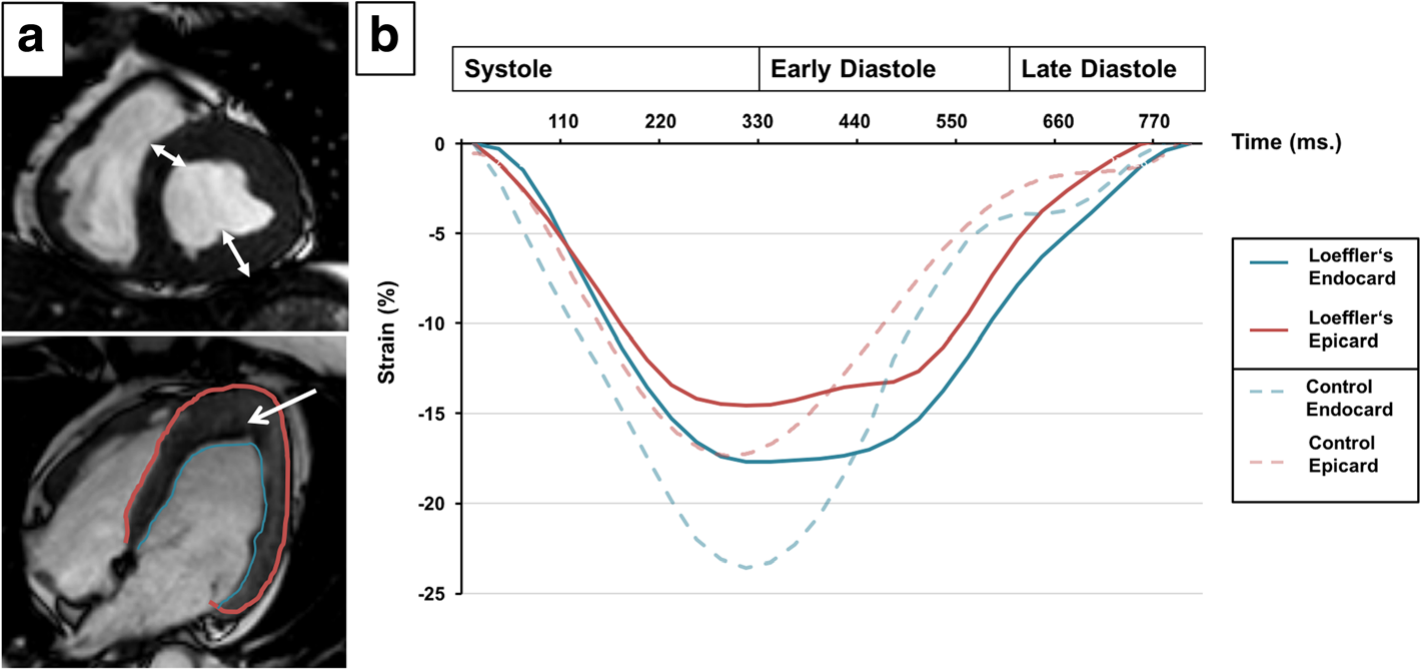
*Functional characteristics of Loeffler’s endocarditis.* **a** Cine steady state free precession images (SSFP) in two and four chamber views. Thickened myocardial wall is indicated by doubled arrows. Altered myocardium in terms of subendocardial susceptibility artefacts can be seen in native contrast (*arrow*). The blue line marks the endocardial, the red line the epicardial contour used for myocardial strain analysis. **b** Systolic endocardial and epicardial global longitudinal strains (GLS) were altered compared to literature standards and an age-matched control (*dotted blue line* vs. continuous *blue line* for endocardial GLS; *dotted red line* vs. continuous *red line* for epicardial GLS). Epi- and endocardial borders revealed diastolic dysfunction in terms of delayed diastolic relaxation

Cine steady state free precession (SSFP) images revealed normal left and right ventricular function (LV ejection fraction (EF) 61%, RV-EF 61%). As can be seen in Fig. 1a (*doubled arrows*), LV and RV walls were thickened and displayed subendocardial susceptibility artefacts (arrow). Despite normal LV-EF, myocardial feature tracking analysis (TomTec Imaging Systems, Unterschleißheim, Germany) revealed impaired systolic endocardial and epicardial global longitudinal strain (GLS) compared to literature standards (-17.7% vs. -22.2% for endocardial GLS; -14.5% vs. -20.4% for epicardial GLS) [6]. Peak early diastolic strain rate (SRe) was impaired in epi- and endocardium as well, indicating diastolic impairment (1.2 s^-1^ vs. 1.7 s^-1^ for endocardial diastolic SRe; 1.0 s^-1^ vs. 1.5 s^-1^ for epicardial diastolic SRe) (Fig. 1b).

**Fig. 2:**
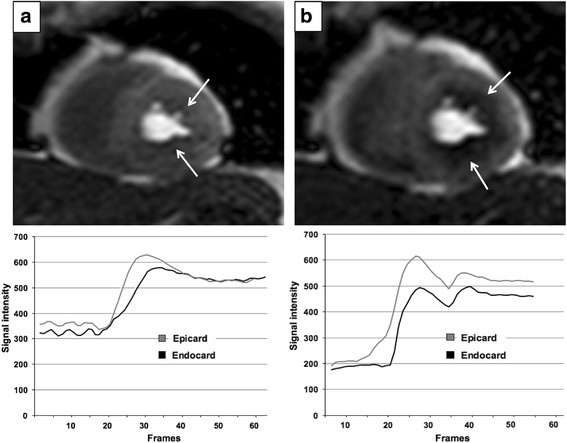
*Coronary flow characteristics of Loeffler’s endocarditis.* Myocardial perfusion at rest (**a**) and in hyperaemia state (**b**). At rest, a small lack of endocardial perfusion is present (*arrows*, **a**). The endocardial intensity-time curve shows a slower contrast agent uptake in comparison to the epicardial curve still reaching epicardial intensity at the end of the sequence. This phenomenon was intensified in regadenoson stress protocol (*arrows*, **b**). Endocardial intensity-time curve did not reach epicardial values throughout the sequence indicating impaired coronary microcirculation

Perfusion images showed an imbalance of transmyocardial perfusion already under resting conditions (Fig. 2a, *arrows*). This phenomenon was aggravated during regadenoson induced hyperaemia, that showed highly impaired perfusion in endocardial layers, indicating altered coronary microcirculation (Fig. 2b).

**Fig. 3:**
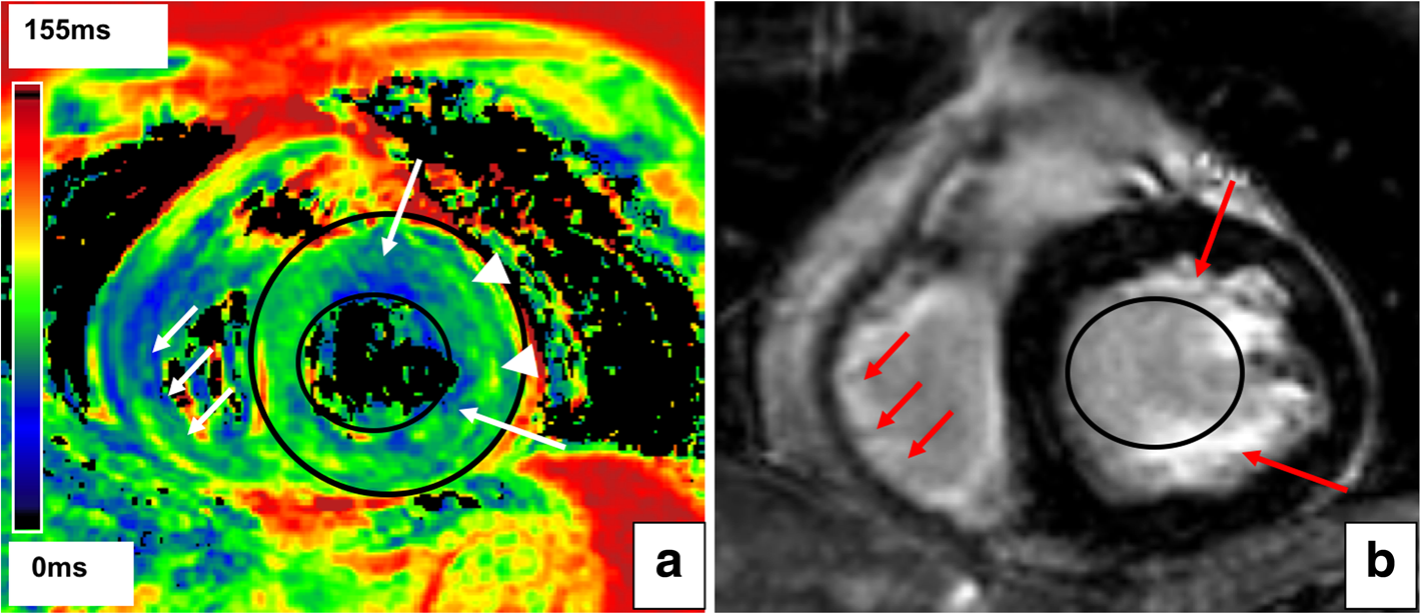
*Myocardial texture of Loeffler’s endocarditis.* **a** Parametric T2 mapping revealed a slightly reduced T2 time in subendocardial layers (*white arrows*). Interestingly, epicardial T2 time was elevated (*arrowheads*) indicative for acute tissue oedema. **b** Late gadolinium enhancement (LGE) in subendocardial layers (*red arrows*) of the left and right ventricle corresponded to reduced T2 time suspective of subendocardial fibrosis. However, epicardial layers were not affected with LGE, but with increased T2 time

To analyse myocardial tissue texture, a parametric CMR was conducted to separate oedema or acute necrosis to other tissue texture components with T2 mapping [7]. As shown in Fig. 3a, T2 mapping revealed a slightly reduced T2 time (55.0 ± 8.4 ms, arrows) compared to an age- and gender-matched control (56.4 ± 3.5), which was still in the normal value range according to current literature [8]. Epimyocardial T2 time was elevated compared to the control (69.6 ± 11.9 ms vs. 60.3 ± 4.6 ms, arrowheads) suspecting acute tissue oedema [8]. Diffuse subendocardial late gadolinium enhancement (LGE) was detected in both chambers in those areas of slightly reduced T2 time completing the previous findings (Fig. 3b, *arrows*).

Taking the localisation of fibrosis, the T2 map and the perfusion abnormalities into account, the hypotheses of vascular coronary disease or myocarditis were discarded since there was neither an endocardial lesion corresponding to a coronary territory nor an epicardial lesion. Additional details of the patient’s history revealed a hypereosinophilia (max. 11.900/μl, normal value < 440/μl) for more than 6 months. On the basis of the CMR findings and the patient’s history of T-NOS with hypereosinophilia, he was diagnosed to have Endocarditis parietalis fibroplastica Löfflein (EPF). Interestingly, the multiparametric CMR workup revealed different stages of myocardial involvement: reduced GLS, diffuse LGE, perfusion defect and slightly lower T2-time displayed fibrosis and reduced coronary flow of the endocardium already under resting conditions. The epicardium, however, was characterized by abnormal GLS with abnormal diastolic function, lack of LGE, preserved perfusion, but elevated T2 times suggesting an inflammatory stadium. A high-dose corticoid drug (Dexamethasone 40 mg) was added to the existing everolimus-therapy and the patient was transferred to the department of haematology for further diagnostics. His bone marrow showed elevated numbers of eosinophilia up to 10% during completion of diagnostic steps.

Cardiac involvement at different stages of EPF as shown by multiparametric CMR characterized T-NOS as a palliative situation, as recent therapeutic regimes such as immunosuppressive drugs and stem cell transplantation did not lead to a lasting remission.

Since multiparametric CMR workup could not only identify EPF but also characterize the patchy disease state (T2 map, LGE and myocardial strain) and stratify the patient’s individual prognosis, invasive coronary angiography and endomyocardial biopsy were not conducted.

## Discussion and Conclusion

The present case of EPF illustrates that multiparametric CMR can not only identify EPF, but also characterize EPF according to the disease state. CMR is a highly efficient tool for the process of diagnosis if invasive procedures are discarded due to risk/benefit assessment. In this particular case, myocarditis and coronary artery disease could be excluded without EMB or angiography due to multiparametric CMR. In addition, CMR detected advanced endocardial fibrosis with wall thickening as morphological hallmarks of the disease, while those features would have technically impeded adequate EMB.

## Abbreviations

CMR: Cardiac magnetic resonance
ECG: Electrocardiogram
EF: Ejection fraction
EMB: Endomyocardial biopsy
EPF: Endocarditis parietalis fibroplastica Löfflein
GLS: Global longitudinal strain
HES: Hypereosinophilic syndromes
IL-5: Interleukin 5
LGE: Late gadolinium enhancement
LV: Left ventricle
RV: Right ventricle
SSFP: Steady state free precession
T-NOS: Non-specified T-cell lymphoma
TTE: Transthoracic echocardiography
